# An Intelligent Load Control-Based Random Access Scheme for Space-Based Internet of Things

**DOI:** 10.3390/s21041040

**Published:** 2021-02-03

**Authors:** Changjiang Fei, Bin Jiang, Kun Xu, Lei Wang, Baokang Zhao

**Affiliations:** 1 College of Information and Communication, National University of Defense Technology, Wuhan 430010, China; feichangjiang.hi@163.com (C.F.); xukunown@163.com (K.X.); wanglei_nudt@163.com (L.W.); 2 College of Computer, National University of Defense Technology, Changsha 410073, China; bkzhao@nudt.edu.cn

**Keywords:** random access, space-based Internet of Things, Internet of Things, artificial neural networks, support vector machines

## Abstract

Random access is one of the most competitive multiple access schemes for future space-based Internet of Things (S-IoT) due to its support for massive connections and grant-free transmission, as well as its ease of implementation. However, firstly, existing random access schemes are highly sensitive to load: once the load exceeds a certain critical value, the throughput will drop sharply due to the increased probability of data collision. Moreover, due to variable satellite coverage and bursty traffic, the network load of S-IoT changes dynamically; therefore, when existing random access schemes are applied directly to the S-IoT environment, the actual throughput is far below the theoretical maximum. Accordingly, this paper proposes an intelligent load control-based random access scheme based on CRDSA++, which is an enhanced version of the contention resolution diversity slotted ALOHA (CRDSA) and extends the CRDSA concept to more than two replicas. The proposed scheme is dubbed load control-based three-replica contention resolution diversity slotted ALOHA (LC-CRDSA3). LC-CRDSA3 actively controls network load. When the load threatens to exceed the critical value, only certain nodes are allowed to send data, and the load is controlled to be near the critical value, thereby effectively improving the throughput. In order to accurately carry out load control, we innovatively propose a maximum likelihood estimation (MLE)-based load estimation algorithm, which estimates the load value of each received frame by making full use of the number of time slots in different states. On this basis, LC-CRDSA3 adopts computational intelligence-based time series forecasting technology to predict the load values of future frames using the historical load values. We evaluated the performance of LC-CRDSA3 through a series of simulation experiments and compared it with CRDSA++. Our experimental results demonstrate that in S-IoT contexts where the load changes dynamically, LC-CRDSA3 can obtain network throughput that is close to the theoretical maximum across a wide load range through accurate load control.

## 1. Introduction

The goal of the Internet of Things (IoT) is to build a world in which everything is connected. Due to the coverage limitations of terrestrial networks such as the Internet and mobile communication networks, it is difficult for terrestrial IoT to provide services to areas lacking ground infrastructure, such as forests, deserts and oceans. Space-based information networks have key and significant advantages, including global coverage, independent infrastructure and strong anti-destructive ability, and will thus play a vital role in future IoT. The space-based Internet of Things (S-IoT) [1,2,3,4,5,6,7] uses the space-based information network for IoT information transmission, and has accordingly attracted the attention of many organizations, including Globalstar, Inmarsat, Iridium and Orbcomm. The S-IoT is an extension to and supplement of the terrestrial IoT and is used primarily to connect nodes in areas that are difficult for terrestrial IoT to cover, such as oceans [8], polar regions [9] and deserts, as well as nodes in areas posing unique challenges, such as disaster areas and battlefields.

In S-IoT, uplink multiple access is a key technology that supports massive ground nodes in efficiently reporting data to satellites. However, the uplink multiple access design of S-IoT also presents significant challenges. First, in S-IoT, a single satellite typically covers thousands of kilometers and is required to provide data transmission services for massive ground nodes. By 2025, the number of machine-to-machine (M2M) and IoT networks connected to space-based information networks is expected to reach 5.96 million [10]. Accordingly, the uplink multiple access scheme in S-IoT needs to support massive connections. Second, the data transmitted through S-IoT are usually short packets with small payloads, such as sensor values and radio frequency identification (RFID) tag information. If nodes obtain channel resources in a request-grant manner, this will introduce massive signaling overhead. This is unaffordable for S-IoT, as satellite bandwidth is typically low and the uplink and downlink bandwidth is highly asymmetric; accordingly, the uplink multiple access of S-IoT must be grant-free. Third, in S-IoT, ground nodes are usually distributed in the wild and may even be carried by animals. Due to limitations resulting from the weight, volume and deployment environment, the computing, storage and energy resources of ground nodes are severely limited, which entails that the operations in ground nodes must be simple enough to function within these limitations.

Among the existing multiple access schemes, random access can readily meet the above challenges, making it one of the most competitive multiple access schemes for future S-IoT applications. We will introduce and analyze existing multiple access schemes in detail in Section 2. In random access, after a ground node generates data, it sends them directly to the satellite according to certain rules without the need to interact with the satellite or allocate channel resources in advance. In short, random access supports the sharing of satellite channels by massive nodes, operates in a grant-free manner, and is easy to implement in ground nodes.

However, serious problems arise when the existing random access schemes are applied directly to S-IoT. Below we specifically introduce our analysis of these problems. First and foremost, existing random access schemes are highly sensitive to load. When the load exceeds a certain critical value, the throughput will drop sharply due to the increased probability of data collision. For example, when the normalized load is 0.65 (packets/slot), contention resolution diversity slotted ALOHA (CRDSA) [11] achieves a maximum throughput of about 0.52 (packets/slot). When the normalized load is 1, however, the throughput of CRDSA is only about 0.36.

Moreover, due to the variable nature of satellite coverage and the burstiness of its traffic, the network load of S-IoT changes dynamically. For example, when the orbit altitude is 500 km and the half-beam direction angle is 60°, due to satellite movement, the satellite coverage area changes by about 840,000 km^2^ every minute, with an change ratio of about 27%. Due to the uneven distribution of ground nodes, the number of nodes covered by satellites varies drastically. In addition, in a given period of time (such as a slot or a frame), only a small proportion of nodes have data to transmit, while most of the nodes are in a sleep state. In adjacent time periods, the number of nodes that need to send data may fluctuate drastically, which gives uplink traffic its strongly bursty nature.

In the process of dynamic load changes, when the load is low, although the probability of data collisions is small, the throughput is low due to few packets being transmitted; when the load is high and exceeds the critical value, however, the packet loss rate (PLR) increases sharply due to the increased probability of data collisions, meaning that high throughput also cannot be obtained under these circumstances. Worse yet, packets that suffer a failed transmission (hereinafter referred to as failed packets) will need to be retransmitted at a later point in time; this results in the accumulation of packets within the network, increasing the frequency at which the load exceeds the critical value and further reducing the throughput. This problem is further exacerbated when the average load is high. Too many packets accumulating in the network may even result in network paralysis. It is therefore difficult for existing random access schemes to be adapted to scenarios with dynamic load changes, while the actual throughput is also much lower than the theoretical maximum.

One direct but highly effective method of enabling random access to obtain higher throughput in S-IoT with dynamic load changes is that of controlling the network load. When the load threatens to exceed the critical value, only some nodes are allowed to send data, and the load is controlled to be close to the critical value. Data that has no chance of being transmitted due to load control will be transmitted later. This method adopts the concept of “peak-cutting and valley-filling” to stabilize the load and significantly improve throughput. First, it avoids the decrease in throughput when the load exceeds the critical value. Moreover, it can increase the load of frames whose load is originally lower than the critical value, thereby increasing throughput.

When applying this method, the core problem to be solved is that of how to determine the future load. One apparently intuitive solution would be to use historical load values to predict future load values. However, due to data collision, satellites usually cannot successfully receive all packets, meaning that we typically do not know how many packets are sent by the ground nodes in reality. In order to obtain the historical load values, it is necessary to estimate the load of each received frame. Building on this concept, this paper proposes an intelligent load control-based random access scheme based on CRDSA++ [12] (see Section 2.3.2 for details), which is an enhanced version of the CRDSA and extends the CRDSA concept to more than two replicas. The proposed scheme is dubbed load control-based three-replica contention resolution diversity slotted ALOHA (LC-CRDSA3). The contributions of this paper can be summarized as follows:

- To the best of our knowledge, this paper is the first in the field of random access to improve throughput under a dynamically changing load. The proposed LC-CRDSA3 scheme is highly suitable for S-IoT, and obtains network throughput that approaches the theoretical maximum across a wide load range under dynamic load.
- In order to accurately implement load control, we innovatively propose a maximum likelihood estimation (MLE)-based load estimation algorithm, which estimates the load by making full use of the number of time slots in different states. On this basis, we explore the use of computational intelligence-based time series forecasting technology for load forecasting.
- The proposed LC-CRDSA3 scheme is verified through a series of simulation experiments and compared with CRDSA++.

The remainder of this paper is organized as follows: Section 2 reviews the work related to multiple access. Section 3 elaborates the LC-CRDSA3 scheme proposed in this paper, including the overall LC-CRDSA3 framework and the specific load estimation and load forecasting processes. Section 4 evaluates the performance of the proposed LC-CRDSA3. Finally, Section 5 draws conclusions and proposes future work.

## 2. Related Work

At present, only a small amount of research on multiple access has been conducted in the S-IoT context, and these studies are based primarily on existing multiple access schemes. A large number of multiple access schemes have been proposed in the context of space-based information networks and terrestrial networks. These schemes can be broadly divided into three categories: orthogonal multiple access, non-orthogonal multiple access and random access. We introduce these three types of algorithms in the three respective subsections below.

### 2.1. Orthogonal Multiple Access

Orthogonal multiple access is a type of traditional and classic multiple access scheme. It is based on the theory of channel division, which involves dividing the wireless link into multiple orthogonal channels, such that each user transmits data through one channel. Typical orthogonal multiple access schemes include frequency division multiple access (FDMA), time division multiple access (TDMA), code division multiple access (CDMA), etc.

Orthogonal multiple access enables data collisions to be avoided entirely, as the channels are orthogonal to each other. However, serious problems arise when orthogonal multiple access schemes are applied to S-IoT. First, due to channel resource limitations, orthogonal multiple access can provide only a very limited number of channels, making it difficult to support massive connections. Moreover, the ground nodes in S-IoT only have data to transmit in very short time periods and are in a sleep state most of the time; thus, allocating a channel to each node would result in a serious waste of channel resources. One way to reduce channel resource wastage and increase user capacity would be to allocate channels according to data transmission needs. This is the approach adopted by demand assignment multiple access (DAMA) scheme [13]. Under this scheme, however, the ground nodes are required to obtain channel resources through request-grant processes; as discussed above, since the ground nodes in this context mainly send short packets, it would be difficult for S-IoT to accommodate the signaling overhead caused by the request-grant process.

### 2.2. Non-Orthogonal Multiple Access

In order to overcome the above-mentioned problems of orthogonal multiple access, a series of non-orthogonal multiple access (NOMA) schemes have been proposed for massive machine-type communication (mMTC) [14] in the fifth-generation mobile communication (5G) context [15]. In NOMA, through multiplexing of e.g., the power and code domains, users can share the same channel resources (such as time and frequency). Typical NOMA schemes include power domain non-orthogonal multiple access (PD-NOMA) [16], interleave division multiple access (IDMA) [17], resource spread multiple access (RSMA) [18], multi-user shared access (MUSA) [19] and sparse code multiple access (SCMA) [20], among others. These NOMA schemes are multiplexed from the domains of transmitting power, interleaver, sequence, and codeword.

Through the non-orthogonal division of resources, NOMA can significantly increase the network user capacity. NOMA could therefore be used as a candidate multiple access scheme for S-IoT. While some studies have attempted to apply NOMA to S-IoT, these works mainly solve certain specific problems in its application, including beamforming [21], downlink resource allocation [22], and robust design [23]. An asynchronous flipped SCMA scheme was proposed in [24]. By introducing the concept of flipped diversity into SCMA, this scheme reduces the probability of codebook collisions by transmitting data packets and their flipped replicas simultaneously, thereby improving system performance.

However, there are still some problems associated with applying NOMA to S-IoT. First, although NOMA can increase user capacity, it is difficult for it to do so to an extent that meets the needs of the S-IoT environment. The user capacity of NOMA schemes can be several times that of orthogonal multiple access. In 5G, a base station usually covers several hundred meters and connects tens to hundreds of nodes; in S-IoT, however, the coverage of a satellite (equivalent to a base station) extends over thousands of kilometers, while the number of connected nodes can reach tens of thousands or even more. Second, because the channel resources used by different users are not completely orthogonal, interference between users will arise. To solve this problem, the receiver usually adopts receiving processing techniques such as successive interference cancellation (SIC). When using SIC technology, the receiver demodulates the signals of each user one by one in a specific order. When the number of users is large, the reception processing on satellites will become very complicated.

### 2.3. Random Access

Since no request-grant process is required, random access is highly suitable for scenarios involving short and bursty data packets. In S-IoT, Zhen et al. [25] studied the reliable design and detection of random access preamble to enhance the access efficiency in low earth orbit (LEO) scenarios. Random access typically adopts the ALOHA method. The basic concept of random access involves sending data regardless of whether there is a data collision; if a failed transmission occurs, the packet will be retransmitted later after a period of time has elapsed.

#### 2.3.1. Traditional Random Access

Pure ALOHA (P-ALOHA) was one of the first random access schemes to be proposed. In P-ALOHA, each user sends data immediately after generating it. If a data collision occurs, the user waits for a randomly selected period of time before resending the data to avoid subsequent collision. The maximum throughput of P-ALOHA is only 0.18 [26].

In order to improve the throughput of P-ALOHA, slotted ALOHA (S-ALOHA) [26] was proposed. Unlike P-ALOHA, S-ALOHA divides time into a series of slots, such that data is transmitted in standardized slots. After users generate data, they need to wait until the beginning of the next slot before sending it. S-ALOHA eliminates the problem of head-tail collision in P-ALOHA, thereby reducing the probability of data collision, and its maximum throughput can reach 0.36.

When a user needs to send a long packet, if it is sent via P-ALOHA or S-ALOHA, the process of data collision and retransmission will result in a large transmission delay for this packet. Reservation ALOHA (R-ALOHA) [27] was accordingly proposed to improve long packet transmission performance. In R-ALOHA, when a user needs to send a long packet, they apply for a series of slots from the satellite and send a batch of data. Obviously, R-ALOHA is not suitable for short packet transmission, as the process of applying for slots introduces significant signaling overhead; if packets are very short, this will cause serious resource wastage. Although these traditional random access schemes are simple to implement, their throughput is relatively low due to the high probability of data collision.

#### 2.3.2. Improved Random Access

In order to further improve the throughput of random access, researchers have made a number of improvements to traditional random access schemes. Some representative improved schemes are introduced below.

Diversity slotted ALOHA (DSA) [28] is an improved version of S-ALOHA. After sending a packet, the user randomly waits for some slots within a frame, then sends the packet again; in this way, the number of transmissions is boosted to increase the probability of successful packet reception. Since increasing the number of transmissions also increases the probability of data collision, however, the throughput improvement of DSA is not obvious.

In contention resolution diversity slotted ALOHA (CRDSA) [11], every packet is sent twice in different slots of a frame; i.e., every packet is represented by two replicas in a frame. The receiver uses SIC technology to decode the data. More specifically, in a round of iteration, the receiver first decodes the packets in the slots without collisions, then deletes the replicas of these packets transmitted in other slots to eliminate interference. The receiver repeats this process until either no new packets can be decoded or the upper limit of the number of iterations is reached. It should be noted that when SIC technology is used in random access, all packets without collisions can be decoded in one iteration, instead of all packets sent by one user. Therefore, the implementation complexity of SIC will not rapidly increase as the number of users increases. Due to the full utilization of the colliding packets through SIC technology, CRDSA can significantly increase throughput, with a maximum throughput of around 0.52.

In order to further enhance throughput, CRDSA++ [12] improves CRDSA in two key ways: by increasing the number of replicas of each packet (three to five replicas), and by further decoding packets through leveraging the signal power difference between different users. These innovations boost CRDSA++’s throughput significantly compared to CRDSA. When the number of replicas of each packet is 3, CRDSA++ achieves a maximum throughput of about 0.7 for a normalized load of 0.7 [29]; that is, the critical load value of CRDSA++ is 0.7 for three-replica methods.

Other improved random access schemes include irregular repetition slotted ALOHA (IRSA) [30], coded slotted ALOHA (CSA) [31], rateless multiple access (RMA) [32] and grant-free RMA [33] etc. Although these schemes can improve throughput to a certain extent, they also increase implementation complexity and are thus difficult to apply to S-IoT.

As mentioned in Section 1, random access has the key and significant advantages of supporting massive connections and grant-free transmission, as well as simple implementation. This paper tackles the problem of the actual throughput of existing random access schemes in S-IoT with a highly dynamic load being much lower than the theoretical maximum.

CRDSA++ is a classic random access scheme in space-based information networks. It has a relatively high throughput and is very easy to implement. CRDSA++ obtains the highest peak throughput when each packet has three replicas [29]. Therefore, the scheme proposed in this paper is based on three-replica CRDSA++ (which we refer to as CRDSA3). Notably, the load control concept proposed in this paper can also be applied to other load-sensitive multiple access schemes in order to improve their throughput in a network environment with dynamic load.

## 3. LC-CRDSA3: Load Control-Based Three-Replica Contention Resolution Diversity Slotted ALOHA

In this section, we first present the overall framework of the LC-CRDSA3 scheme, then provide a more detailed outline of the basic load estimation and load forecasting processes in our proposed scheme.

### 3.1. Overview

As discussed in Section 1, the key concept underpinning LC-CRDSA3 is that of dynamically controlling the network load. When the load threatens to exceed the critical value, only certain nodes are allowed to send data, and the load is controlled to be close to the critical value, which effectively improves the throughput.

**Figure 1:**
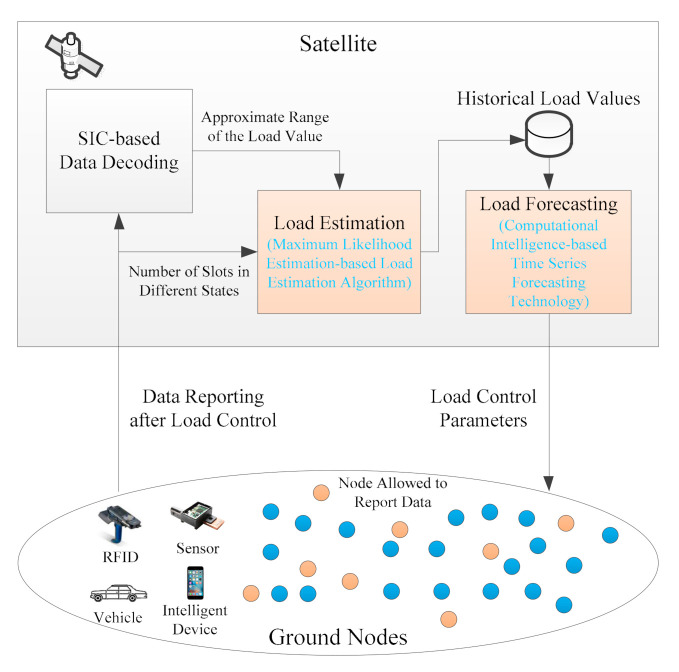
The framework of the load control-based three-replica contention resolution diversity slotted ALOHA (LC-CRDSA3) scheme.

Figure 1 illustrates the framework of the LC-CRDSA3 scheme. When a frame arrives at the satellite, the satellite decodes the data based on SIC technology. In order to control load, LC-CRDSA3 needs to perform load estimation and load forecasting. After the data reception process for each frame is complete, the satellite estimates the load value of the frame (that is, estimates how many packets are sent by ground nodes in this frame). In terms of load estimation, we first determines the approximate range of the load value according to the decoding result of a frame, and innovatively propose an MLE-based load estimation algorithm, which makes full use of the number of slots in different states to estimate the load. Through load estimation, we can obtain the load value of each frame, which is the historical load value. Based on these historical load values, LC-CRDSA3 employs computational intelligence-based time series forecasting technology to forecast the load values of future frames.

If the forecasted load value of a future frame does not exceed the critical value, no load control is required and the ground nodes send data normally. Otherwise, the load needs to be controlled. The satellite calculates the load control parameter according to the forecasted load value, then broadcasts it to the ground nodes before the start of the frame. The load control parameter is the proportion of nodes that are permitted to send data, and also represents the probability of each node being able to send data. After receiving the load control parameter, the ground nodes determine whether to send data according to the parameter in the corresponding frame. If the load forecasting is sufficiently accurate, the load after controlling will be near the critical value, thereby avoiding the throughput drop caused by excessive load.

### 3.2. Load Estimation

Even if SIC technology is employed, a satellite usually cannot decode all packets transmitted in a frame. After the data decoding process is complete, some packets may still remain in some slots that cannot be decoded due to the presence of some so-called “loops” [11]. Due to data collisions, we cannot know exactly how many packets remain in these slots. Accordingly, in order to obtain the historical load value, when the satellite is unable to decode all packets in a frame, load estimation is required.

Suppose that the load value of a particular frame (that is, the number of packets transmitted in this frame) is L, the number of replicas of each packet is r, and the number of slots contained in a frame is M. According to the number of packets (or packet replicas, when r>1) in a slot, each slot has three states: idle (the number of packets is 0), successful (the number of packets is 1) and collided (the number of packets exceeds 1). Accordingly, we refer to slots in these three states as idle slots, successful slots, and collided slots respectively. The number of idle, successful and collided slots in a frame is denoted by i, s, and c, respectively. Since the number of slots in different states will be affected by L, we can estimate L by observing the values of i, s, and c in the frame. The estimated value of L is represented by $\hat{L}$. We define the load estimation precision of a frame as follows:

(1) $$A^{E}=1-\frac{\left|\hat{L}-L\right|}{L}.$$

The load estimation precision will affect the accuracy of subsequent load forecasting.

For not sending multiple replicas (that is, when r=1), several load estimation algorithms have been proposed. Vogt [34] proposed a simple load estimation algorithm, which assumes that each collided slot contains two packets; its load estimation expression is:

(2) $${\hat{L}}^{\mathrm{Vog}}=s+2c.$$

In [35], Cha et al. modified the above algorithm, assuming that each collided slot contains 2.39 packets on average, i.e.,:

(3) $${\hat{L}}^{\mathrm{Cha}}=s+2.39c.$$

Another load estimation algorithm was proposed by Khandelwal et al. [36]; this algorithm uses only the number of idle slots to estimate the load value. The load estimation expression is as follows:

(4) $${\hat{L}}^{\mathrm{Kha}}=\arg\underset{L}{\min}\left|E\left(I\right)-i\right|=\lceil \frac{\mathrm{lg}\left(\frac{M}{i}\right)}{\mathrm{lg}\left(\frac{M}{M-1}\right)}\rfloor ,$$

where I represents the random variable of the number of idle slots in a frame, E(I) is the expectation of I, and ⌈ · ⌋ represents integer rounding. When r=1:

(5) $$E\left(I\right)=M{\left(1-\frac{1}{M}\right)}^{L}.$$

None of the above algorithms make full use of the number of slots in the three different states for load estimation, and the algorithm in Equation (4) is not suitable for situations in which no idle slot exists. In order to further improve the load estimation precision, we propose an MLE-based load estimation algorithm. This algorithm first determines the approximate range of the load value according to the decoding result of a frame, then uses MLE to accurately estimate the load.

Suppose the number of packets successfully received in a certain frame is *L*^S^, while the number of slots still in a collided state following data decoding is $c^{\prime }$. Each packet will generate r replicas, and there are at least two packet replicas in each collided slot; thus:

(6) $$L\geq L^{S}+\left\lceil \frac{2c^{\prime }}{r}\right\rceil .$$

We use I, S, and C to represent the random variables of the number of idle slots, successful slots and collided slots, respectively. Taking the idle slots as an example, according to the basic concept of MLE, since a sample $I_{1}$ of the random variable I has already taken the sample value i, we contend that the probability $p^{I}(i;L)$ of $I_{1}$ taking this sample value is relatively large. $p^{I}(i;L)$ will be affected by L; therefore, the value of L needs to make $p^{I}(i;L)$ relatively large. If only the sample value i is used to estimate L, we can obtain the estimated value:

(7) $${\hat{L}}^{I}=\arg\underset{L\geq L_{\min}}{\max}p^{I}(i;L),$$

in which $L_{\min}=L^{S}+\left\lceil 2c^{\prime }/r\right\rceil$.

Suppose that the probability of samples $S_{1}$ and $C_{1}$ of random variables S and C taking sample values s and c is $p^{S}(s;L)$ and $p^{C}(c;L)$ respectively. In the same way, the value of L also needs to make $p^{S}(s;L)$ and $p^{C}(c;L)$ relatively large. Therefore, we choose the $\hat{L}$ that maximizes $p^{I}(i;L)\cdot p^{S}(s;L)\cdot p^{C}(c;L)$ as the estimated value of L; that is:

(8) $$\hat{L}=\arg\underset{L\geq L_{\min}}{\max}\left\{p^{I}(i;L)\cdot p^{S}(s;L)\cdot p^{C}(c;L)\right\}.$$

As is clear from the above, we use the number of slots in the three states simultaneously to estimate the load. It should be noted that, unlike the general MLE, we use the sample values of three random variables to calculate the estimated value of the parameter; accordingly, although there is only one sample value for each random variable, we can still obtain high estimation precision.

We next derive the expressions of $p^{I}(i;L)$, $p^{S}(s;L)$ and $p^{C}(c;L)$. As mentioned earlier, a packet $p_{l}$ (l=1,⋯,L) generates r replicas, which randomly select r different slots from a total of M slots for transmission. The probability of a slot $S_{m}$ (m=1,⋯,M) being selected by the packet $p_{l}$ is thus:

(9) $$p\left\{p_{l}\in S_{m}\right\}=\frac{r}{M}.$$

Each packet selects slots independently. If slot $S_{m}$ is in the idle state, this means no packet has selected it; the corresponding probability is:

(10) $$p^{i}={\left\{1-p\left\{p_{l}\in S_{m}\right\}\right\}}^{L}={\left(1-\frac{r}{M}\right)}^{L}.$$

When slot $S_{m}$ is selected by only one packet, it is in the successful state, the corresponding probability of which is:

(11) $$p^{s}=C_{L}^{1}\cdot p\left\{p_{l}\in S_{m}\right\}\cdot {\left\{1-p\left\{p_{l}\in S_{m}\right\}\right\}}^{L-1}=\frac{Lr}{M}{\left(1-\frac{r}{M}\right)}^{L-1}.$$

Hence, the probability of slot $S_{m}$ being in the collided state is:

(12) $$p^{c}=1-p^{i}-p^{s}=1-{\left(1-\frac{r}{M}\right)}^{L}-\frac{Lr}{M}{\left(1-\frac{r}{M}\right)}^{L-1}=1-\frac{M+Lr-r}{M}{\left(1-\frac{r}{M}\right)}^{L-1}.$$

Therefore:

(13) $$p^{I}(i;L)=C_{M}^{i}\cdot {\left(p^{i}\right)}^{i}\cdot {\left(1-p^{i}\right)}^{M-i}=C_{M}^{i}{\left(1-\frac{r}{M}\right)}^{iL}{\left[1-{\left(1-\frac{r}{M}\right)}^{L}\right]}^{M-i},$$

(14) $$p^{S}(s;L)=C_{M}^{s}\cdot {\left(p^{s}\right)}^{s}\cdot {\left(1-p^{s}\right)}^{M-s}=C_{M}^{s}{\left[\frac{Lr}{M}{\left(1-\frac{r}{M}\right)}^{L-1}\right]}^{s}{\left[1-\frac{Lr}{M}{\left(1-\frac{r}{M}\right)}^{L-1}\right]}^{M-s},$$

(15) $$p^{C}(c;L)=C_{M}^{c}\cdot {\left(p^{c}\right)}^{c}\cdot {\left(1-p^{c}\right)}^{M-c}=C_{M}^{c}{\left[1-\frac{M+Lr-r}{M}{\left(1-\frac{r}{M}\right)}^{L-1}\right]}^{c}{\left[\frac{M+Lr-r}{M}{\left(1-\frac{r}{M}\right)}^{L-1}\right]}^{M-c}.$$

The above load estimation process estimates the number of packets actually sent in a frame. When implementing load control, it is necessary to forecast how many packets need to be sent before load control in future frames. We refer to the number of packets that need to be sent before load control in a frame as the original load of the frame. Therefore, in subsequent load forecasting, we need to forecast the original load values of future frames based on the historical values of the original load. If load control has been conducted in a frame, and the corresponding load control parameter is *p*^LC^ (this is known at the time of load estimation), then the estimated value of the original load of the frame is:

(16) $${\hat{L}}^{\prime }=\frac{\hat{L}}{p^{\mathrm{LC}}}$$

Otherwise, the estimated value of the actual load of the frame is the estimated value of the original load; that is:

(17) $${\hat{L}}^{\prime }=\hat{L}.$$

### 3.3. Load Forecasting

It is first necessary to determine whether the load needs to be controlled according to the forecasted load values, after which the load control parameters can be calculated as required. We use time series forecasting technology to forecast the original load values of future frames based on the time series composed of the historical values of the original load in past frames (referred to as the load series). Suppose that the original load value of a frame is $L^{\prime }$, and the forecasted value of $L^{\prime }$ is $L^{F}$. Similarly, we define the load forecast precision of this frame as follows:

(18) $$A^{F}=1-\frac{\left|L^{F}-L^{\prime }\right|}{L^{\prime }}.$$

In the network, a frame’s original load will be affected by the number of newly generated packets in the frame, the number of packets with no chance of being transmitted in the previous frames, the number of retransmitted packets, and the accuracy of the previous load forecasting. Worse yet, since the load series will be affected by the load forecasting process, before a load forecasting method is applied, we cannot obtain the load series in advance under this method for feature analysis. It is therefore difficult to use classic time series models, such as the autoregressive integrated moving average (ARIMA) model, to model the load series.

Computational intelligence-based time series forecasting is a new type of time series forecasting technology. The key advantage of this approach is that it is well-suited to capture the characteristics of the load series through learning. Moreover, we can use the load series during network operation to train the forecasting model dynamically, which enables the model to dynamically adapt to the law of load changes. Two representative and most commonly used methods of this type are neural network-based and support vector machine-based time series forecasting.

Neural network-based time series forecasting is a non-parametric method that makes no assumptions about the time series model. As long as the neural network has enough hidden layers and each hidden layer has enough neurons, this approach can approximate any complex function with any level of precision. However, neural network-based time series forecasting is difficult to guarantee accuracy when the number of learning samples is small; when the number of samples is large, the generalization performance is low.

Support vector machine is an algorithm based on the principle of structural risk minimization. Support vector machine-based time series forecasting is a convex quadratic optimization problem, which can ensure that the extremal solution obtained is the global optimal solution. However, when the number of samples is large, this method causes great computational and storage overhead. Moreover, this method is very sensitive to the parameters and kernel function.

It can be seen that these two methods have their own advantages and disadvantages. Since we do not know the characteristics of the load series, and the characteristics will be affected by the forecasting methods, we explore the use of these two methods for load forecasting respectively.

#### 3.3.1. Neural Network-Based Load Forecasting

**Figure 2:**
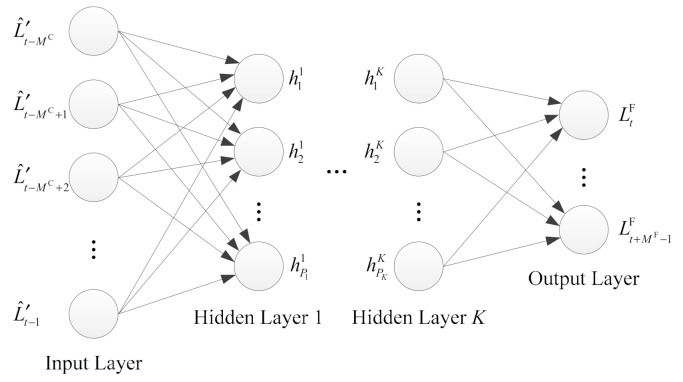
Load forecasting using multilayer feedforward neural network.

As shown in Figure 2, we adopt the most widely used multilayer feedforward neural network for load forecasting. The neural network consists of one input layer, K hidden layers and one output layer. It is assumed that the load value of a frame is strongly correlated with the load values of the past *M^C^* frames; the value of *M^C^* is related to the specific network scenarios. Therefore, we use the load values of the past *M^C^* frames to forecast the load value of a future frame. If we assume that the satellite is currently receiving the signal of the t-th frame, then the input layer contains *M^C^* neurons, which receive *M^C^* historical load values $\left\{{{\hat{L}}^{\prime }}_{t-M^{C}},{{\hat{L}}^{\prime }}_{t-M^{C}+1},\cdots ,{{\hat{L}}^{\prime }}_{t-1}\right\}$ respectively. It should be noted that since the t-th frame has not been completely received, its load value cannot yet be obtained.

The number of hidden layers and the number of neurons in each hidden layer need to be determined according to specific network scenarios. If the number of hidden layers or the number of neurons in each layer is too small, the neural network may not be able to fully capture the characteristics of the load series; on the other hand, if these values are too high, this may cause overfitting and reduce generalization ability. The neural network processes the information from one layer to the next through the activation function. We denote the input layer as layer 0, ${{\hat{L}}^{\prime }}_{t-M^{C}},{{\hat{L}}^{\prime }}_{t-M^{C}+1},\cdots ,{{\hat{L}}^{\prime }}_{t-1}$ as $h_{1}^{0},h_{2}^{0},\cdots ,h_{P_{0}}^{0}$ respectively; then, $P_{0}=M^{C}$, the hidden layers are from layer 1 to layer K, and the output layer is layer K+1. The value of the $p_{k}$-th ($p_{k}=1,\cdots ,P_{k}$) neuron $N_{p_{k}}^{k}$ in the *k*-th (k=1,⋯,K) layer is:

(19) $$h_{p_{k}}^{k}=f_{p_{k}}^{k}\left(a_{p_{k}}^{k}+\sum_{p_{k-1}=1}^{P_{k-1}}w_{p_{k-1},p_{k}}h_{p_{k-1}}^{k-1}\right),$$

in which $h_{p_{k-1}}^{k-1}$ is the value of the $p_{k-1}$-th ($p_{k-1}=1,\cdots ,P_{k-1}$) neuron $N_{p_{k-1}}^{k-1}$ in the (k−1)-th layer, $w_{p_{k-1},p_{k}}$ is the weight of the connection between $N_{p_{k-1}}^{k-1}$ and $N_{p_{k}}^{k}$, $a_{p_{k}}^{k}$ is the constant deviation, and $f_{p_{k}}^{k}(\cdot )$ is the activation function; moreover, the symbolic logic function is typically used, that is:

(20) $$f_{p_{k}}^{k}\left(x\right)=\frac{e^{x}}{1+e^{x}}.$$

The load control parameter of a frame needs to be broadcast to the ground nodes before the start of the frame. Since both load forecasting and load control parameter transmission take time, the load forecasting of a frame needs to be performed in advance. Suppose that the time required for load forecasting is $T^{\mathrm{LF}}$, while the one-way propagation delay between the satellite and ground node is $T^{\mathrm{SG}}$; thus, the time period for which the load forecasting is advanced relative to the start of a frame is $\beta (T^{\mathrm{LF}}+T^{\mathrm{SG}})$, where β is the safety factor. The number of load forecasting steps is therefore:

(21) $$M^{F}=\left\lceil \frac{\beta \left(T^{\mathrm{LF}}+T^{\mathrm{SG}}\right)}{T^{S}M}\right\rceil +1,$$

where $T^{S}$ is the duration of a slot. Accordingly, the output layer contains $M^{F}$ neurons, where the value of the q-th neuron $N_{q}^{K+1}$—that is, the forecasted load value of the (t+q−1)-th frame is:

(22) $$L_{t+q-1}^{F}=f_{q}^{K+1}\left(a_{q}^{K+1}+\sum_{p_{K}=1}^{P_{K}}w_{p_{K},q}h_{p_{K}}^{K}\right).$$

Here, $h_{p_{K}}^{K}$ is the value of the $p_{K}$-th neuron $N_{p_{K}}^{K}$ in the K-th layer, $w_{p_{K},q}$ is the weight of the connection between $N_{p_{K}}^{K}$ and $N_{q}^{K+1}$, $a_{q}^{K+1}$ is the constant deviation, and $f_{q}^{K+1}(\cdot )$ is the activation function; the linear function is typically used, i.e.:

(23) $$f_{q}^{K+1}\left(x\right)=x.$$

Before using the neural network for load forecasting, it is necessary to train the neural network with the historical load values. We use the load series $\left\{{{\hat{L}}^{\prime }}_{t-M^{T}},{{\hat{L}}^{\prime }}_{t-M^{T}+1},\cdots ,{{\hat{L}}^{\prime }}_{t-1}\right\}$ obtained in the previous time period $T^{T}$ to train the neural network, where $M^{T}$ is the number of frames contained in $T^{T}$. In training, the load series $\left\{{\hat{L^{\prime }}}_{m},{\hat{L^{\prime }}}_{m+1},\cdots ,{\hat{L^{\prime }}}_{m+M^{C}-1}\right\}$ ($m=t-M^{T},t-M^{T}+1,\cdots ,t-M^{C}-M^{F}$) is input, and the load series $\left\{{{\hat{L}}^{\prime }}_{m+M^{C}},{{\hat{L}}^{\prime }}_{m+M^{C}+1},\cdots ,{{\hat{L}}^{\prime }}_{m+M^{C}+M^{F}-1}\right\}$ is output. The trained neural network can be regarded as a nonlinear autoregressive model:

(24) $$\left({{\hat{L}}^{\prime }}_{m+M^{C}},{{\hat{L}}^{\prime }}_{m+M^{C}+1},\cdots ,{{\hat{L}}^{\prime }}_{m+M^{C}+M^{F}-1}\right)=f\left({\hat{L^{\prime }}}_{m},{\hat{L^{\prime }}}_{m+1},\cdots ,{\hat{L^{\prime }}}_{m+M^{C}-1}\right)+u_{t},$$

where f(⋅) is the relationship function obtained through training, while $u_{t}$ is the white noise random disturbance.

#### 3.3.2. Support Vector Machine-Based Load Forecasting

The use of support vector machines for time series forecasting is based on support vector regression. As discussed above, in the load forecasting problem, our goal is to predict the load value of the $\left(t+M^{F}-1\right)$-th frame through the historical load values $\left\{{{\hat{L}}^{\prime }}_{t-M^{C}},{{\hat{L}}^{\prime }}_{t-M^{C}+1},\cdots ,{{\hat{L}}^{\prime }}_{t-1}\right\}$ of the past $M^{C}$ frames. We can obtain the forecasted load value of the $\left(t+M^{F}-1\right)$-th frame through $M^{F}$ single-step forecasting. First, we calculate the forecasted load value $L_{t}^{F}$ of the t-th frame using $\left\{{{\hat{L}}^{\prime }}_{t-M^{C}},{{\hat{L}}^{\prime }}_{t-M^{C}+1},\cdots ,{{\hat{L}}^{\prime }}_{t-1}\right\}$, after which we calculate the forecasted load value $L_{t+1}^{F}$ of the (t+1)-th frame through $\left\{{{\hat{L}}^{\prime }}_{t-M^{C}+1},{{\hat{L}}^{\prime }}_{t-M^{C}+2},\cdots ,{{\hat{L}}^{\prime }}_{t-1},L_{t}^{F}\right\}$. By analogy, $L_{t+M^{F}-1}^{F}$ can be forecasted through $\left\{{{\hat{L}}^{\prime }}_{t+M^{F}-M^{C}-1},{{\hat{L}}^{\prime }}_{t+M^{F}-M^{C}},\cdots ,{{\hat{L}}^{\prime }}_{t-1},L_{t}^{F},\cdots ,L_{t+M^{F}-2}^{F}\right\}$.

To successfully execute the above forecasting process, we aim to obtain the functional relationship between the load values $\left\{{L^{\prime }}_{j},{L^{\prime }}_{j+1},\cdots ,{L^{\prime }}_{j+M^{C}-1}\right\}$ of $M^{C}$ consecutive frames and the load value ${L^{\prime }}_{j+M^{C}}$ of the next frame. Let $x={\left({L^{\prime }}_{j},{L^{\prime }}_{j+1},\cdots ,{L^{\prime }}_{j+M^{C}-1}\right)}^{⊤}$ and $y={L^{\prime }}_{j+M^{C}}$; here, our goal is to get the function $h:R^{M^{C}}\to R$ so that:

(25) $$h\left(x\right)=y.$$

We can learn an approximation $\hat{h}$ of h from the load series $\left\{{{\hat{L}}^{\prime }}_{t-M^{T}},{{\hat{L}}^{\prime }}_{t-M^{T}+1},\cdots ,{{\hat{L}}^{\prime }}_{t-1}\right\}$ obtained in the previous time period $T^{T}$ through support vector regression for load forecasting. $N=M^{T}-M^{C}$ samples $\left\{(x_{1},y_{1}),(x_{2},y_{2}),\cdots ,(x_{N},y_{N})\right\}$ can be obtained from $\left\{{{\hat{L}}^{\prime }}_{t-M^{T}},{{\hat{L}}^{\prime }}_{t-M^{T}+1},\cdots ,{{\hat{L}}^{\prime }}_{t-1}\right\}$, where $x_{n}\in R^{M^{C}}$ (n=1,⋯,N) is an $M^{C}$-dimensional vector, and the definitions of $x_{n}$ and $y_{n}$ are as follows:

(26) $$x_{n}=\left[\begin{array}{l} \begin{array}{c} {{\hat{L}}^{\prime }}_{t-M^{T}+n-1}\\ {{\hat{L}}^{\prime }}_{t-M^{T}+n}\\ \vdots \end{array}\\ {{\hat{L}}^{\prime }}_{t-M^{T}+M^{C}+n-2} \end{array}\right],$$

(27) $$y_{n}={{\hat{L}}^{\prime }}_{t-M^{T}+M^{C}+n-1}.$$

The basic concept of support vector regression is to map the samples to a specific feature space $R^{{\tilde{M}}^{C}}$ through a non-linear mapping ϕ, and linear regression can be performed in this space; that is:

(28) $$\hat{h}\left(x\right)=\omega^{⊤}\phi \left(x\right)+b,\;\phi :R^{M^{C}}\to R^{{\tilde{M}}^{C}},\;\omega \in R^{{\tilde{M}}^{C}},$$

where b is the offset. Support vector regression can tolerate a deviation between $\hat{h}(x_{n})$ and $y_{n}$ smaller than ε. We select the linear insensitive loss $\max\{0,|y_{n}-[\omega^{⊤}\phi (x_{n})+b]|-\varepsilon \}$, which has good sparsity and can ensure the generalization ability of the result. Support vector regression can be formally expressed as:

(29) $$\underset{\omega ,b}{\min}\frac{1}{N}\sum_{n=1}^{N}\max\left\{0,\left|y_{n}-\left[\omega^{⊤}\phi \left(x_{n}\right)+b\right]\right|-\varepsilon \right\}+\frac{N}{2D}\omega^{⊤}\omega .$$

In the above formula, D>0 is the penalty factor, which can balance the complexity of the regression model and the degree of sample fit. The larger the D, the higher the degree of fit and the higher the model complexity.

By using the duality principle, and introducing Lagrangian multipliers and kernel function, the above support vector regression can be transformed into the following optimization problem:

(30) $$\underset{\alpha }{\min}\;\frac{1}{2}\sum_{n_{1},n_{2}=1}^{N}\left(\alpha_{n_{1}}-\alpha_{n_{1}}^{*}\right)\left(\alpha_{n_{2}}-\alpha_{n_{2}}^{*}\right)K\left(x_{n_{1}},x_{n_{2}}\right)+\sum_{n=1}^{N}\alpha_{n}\left(y_{n}+\varepsilon \right)-\sum_{n=1}^{N}\alpha_{n}^{*}\left(y_{n}-\varepsilon \right),\;s.t.\;\sum_{n=1}^{N}\left(\alpha_{n}-\alpha_{n}^{*}\right)=0,\;0\leq \alpha_{n}\leq D,\;0\leq \alpha_{n}^{*}\leq D\;(n=1,\cdots ,N).$$

In the above formula, $\alpha ={\left(\alpha_{1},\alpha_{1}^{*},\alpha_{2},\alpha_{2}^{*},\cdots ,\alpha_{N},\alpha_{N}^{*}\right)}^{⊤}$ is the Lagrangian multiplier vector. $K(x_{n_{1}},x_{n_{2}})=\phi {\left(x_{n_{1}}\right)}^{⊤}\cdot \phi (x_{n_{2}})$ is the kernel function, which can combine the two steps of feature mapping and inner product calculation into one step, maintaining the computational complexity at $O(M^{C})$. There are many kernel functions, which need to meet Mercer conditions. We adopt the most commonly used Gaussian kernel function:

(31) $$K\left(x_{i},x_{j}\right)=e^{-\frac{{\left\|x_{i}-x_{j}\right\|}^{2}}{2\sigma^{2}}},$$

where σ is the Gaussian kernel parameter. At this time:

(32) $$\omega =\sum_{n=1}^{N}\left(\alpha_{n}-\alpha_{n}^{*}\right)\phi \left(x_{n}\right).$$

By solving the above convex quadratic programming problem, we can obtain:

(33) $$\hat{h}\left(x\right)=\sum_{n=1}^{N}\left(\alpha_{n}-\alpha_{n}^{*}\right)K\left(x_{n},x\right)+b.$$

According to the KKT (Karush-Kuhn-Tucker) theorem, by deriving equation:

(34) $$\left\{\begin{array}{l} \varepsilon -y_{n}+\hat{h}\left(x_{n}\right)=0\\ \varepsilon +y_{n}-\hat{h}\left(x_{n}\right)=0 \end{array}\right.,$$

the offset *b* can be solved.

As discussed above, after learning to obtain the function $\hat{h}$, through $M^{F}$-step forecasting, we can obtain the forecasted load value of the $\left(t+M^{F}-1\right)$-th frame:

(35) $$L_{t+M^{F}-1}^{F}=\hat{h}\left(x^{F}\right),$$

where $x^{F}=({{\hat{L}}^{\prime }}_{t+M^{F}-M^{C}-1},{{\hat{L}}^{\prime }}_{t+M^{F}-M^{C}},\cdots ,{{\hat{L}}^{\prime }}_{t-1},L_{t}^{F},\cdots ,L_{t+M^{F}-2}^{F}{)}^{⊤}$.

#### 3.3.3. Carrying Out Load Forecasting in the Network

Load forecasting using neural network- or support vector machine-based time series forecasting technology can be divided into two stages, specifically model training and load forecasting.

Model training: Since the load series will be affected by the load forecasting, we cannot obtain the load series samples in advance to train the forecasting model (neural network or support vector machine). In addition, the law of load change usually differs across different time periods. We accordingly update the forecasting model dynamically during use. More specifically, in the initial period $T^{T}$, no load control is performed, and the load series of this period is obtained to train the forecasting model. The trained forecasting model is then used for load forecasting. After that, the forecasting model is retrained every $T^{T}$. Therefore, the forecasting model can update the parameters according to the change of the load series law, thereby ensuring that high load forecast precision is always maintained. In actual scenarios, we can use two forecasting models, $M_{A}$ and $M_{B}$. Note that, here, $M_{A}$ and $M_{B}$ are the same kind of forecasting model (that is, both $M_{A}$ and $M_{B}$ are either neural networks or support vector machines). While a model $M_{A}$ is in use, the other model $M_{B}$ is retrained using the newly obtained load series; when the training of model $M_{B}$ is complete, we switch to using $M_{B}$ for load forecasting, and vice versa.

Load forecasting: Once a forecasting model has been trained, we can use it for load forecasting. By taking the load series $\left\{{{\hat{L}}^{\prime }}_{t-M^{C}},{{\hat{L}}^{\prime }}_{t-M^{C}+1},\cdots ,{{\hat{L}}^{\prime }}_{t-1}\right\}$ of the past $M^{C}$ frames as input, we can obtain the forecasted load value $\left\{L_{t}^{F},L_{t+1}^{F},\cdots ,L_{t+M^{F}-1}^{F}\right\}$ of the subsequent $M^{F}$ frames. As discussed above, we need the forecasted load value $L_{t+M^{F}-1}^{F}$ of the $\left(t+M^{F}-1\right)$-th frame.

We denote the critical load value of CRDSA3 as $L_{0}$. If $L_{t+M^{F}-1}^{F}>L_{0}$, load control is required for the $\left(t+M^{F}-1\right)$-th frame, and its load control parameter is:

(36) $$p_{t+M^{F}-1}^{\mathrm{LC}}=\frac{L_{0}}{L_{t+M^{F}-1}^{F}}.$$

The process of load forecasting is outlined in Algorithm 1. In this algorithm, t=1,2,⋯ is the frame sequence numbers, $m_{\min}^{F}$ is the sequence number of the frame when the load forecasting is first carried out, $S_{t}^{T}$ is the load series used for forecasting model training in the t-th frame, x is a function used to find the remainder of divided by y, TrainModel(X,Y) is the function that uses the load series Y to train the forecasting model X, and LoadForecast(X,Y) is a function that uses the prediction model X to perform load forecasting through the load series Y.

**Algorithm 1** Load ForecastingInput: ${{\hat{L}}^{\prime }}_{t}$ (t=1,2,⋯), $M^{T}$, $M^{C}$, $M^{F}$
Output: $L_{t+M^{F}-1}^{F}$ ($t=m_{\min}^{F},m_{\min}^{F}+1,\cdots$)Initialize: $m_{\min}^{F}=0$
1. **for each **t∈{1,2,⋯}
**do**
2. **if**
$t\geq M^{T}+1$
3. /* Train the forecasting models every $M^{T}$ frames */
4. **if**
$Mod(t,M^{T})=1$5. $S_{t}^{T}=\{{{\hat{L}}^{\prime }}_{t-M^{T}},{{\hat{L}}^{\prime }}_{t-M^{T}+1},\cdots ,{{\hat{L}}^{\prime }}_{t-1}\}$6. **if**
$Mod(\lceil t/M^{T}\rceil ,2)=0$

7. $M_{A}=TrainModel(M_{A},S_{t}^{T})$8. 
**else**9. 
$M_{B}=TrainModel(M_{B},S_{t}^{T})$10. 
**end if**11. 
**end if**
12. 
/* Use the trained forecasting models for load forecasting */13. 
$S_{t}^{F}=\{{{\hat{L}}^{\prime }}_{t-M^{C}},{{\hat{L}}^{\prime }}_{t-M^{C}+1},\cdots ,{{\hat{L}}^{\prime }}_{t-1}\}$
14. 
**if**
$Mod(\lceil t/M^{T}\rceil ,2)=0$

15. 
**if**
$M_{A}$ has been trained16. 
**if**
$m_{\min}^{F}=0$17. 
$m_{\min}^{F}=t$18. **end if**19. 
$L_{t+M^{F}-1}^{F}=LoadForecast(M_{A},S_{t}^{F})$20. 
**else if**
$\lceil t/M^{T}\rceil >2$
21. 
$L_{t+M^{F}-1}^{F}=LoadForecast(M_{B},S_{t}^{F})$22. 
**end if**
23. 
**else**
24. 
**if**
$M_{B}$ has been trained25. 
$L_{t+M^{F}-1}^{F}=LoadForecast(M_{B},S_{t}^{F})$26. 
**else**27. 
$L_{t+M^{F}-1}^{F}=LoadForecast(M_{A},S_{t}^{F})$28. 
**end if**
29. 
**end if**
30. 
**end if**
31. 
**end for**
32. **return**
$L_{t+M^{F}-1}^{F}$ ($t=m_{\min}^{F},m_{\min}^{F}+1,\cdots$)

#### 3.3.4. The Impact of Load Forecast Precision on Throughput

When the forecasted load value $L^{F}$ of a frame is less than or equal to the critical load value $L_{0}$, no load control is performed, and the load forecasting process will not affect the throughput of the frame. When $L^{F}$ is greater than $L_{0}$, the frame will carry out load control. Suppose the original load of the frame is $L^{\prime }$, while the load forecast precision is $A^{F}$; then, $L^{F}$ is equal to $A^{F}L^{\prime }$ (50% probability) or $(2-A^{F})L^{\prime }$ (50% probability). The load control parameter of this frame is:

(37) $$p^{\mathrm{LC}}=\left\{\begin{array}{l} \frac{L_{0}}{A^{F}L^{\prime }}\;\mathrm{if}\;L^{F}=A^{F}L^{\prime }\\ \frac{L_{0}}{\left(2-A^{F}\right)L^{\prime }}\;\mathrm{otherwise}\; \end{array}\right.,$$

and the load of this frame after load control—that is, the actual load—is:

(38) $$L=\left\{\begin{array}{l} \frac{L_{0}}{A^{F}}\;\mathrm{if}\;L^{F}=A^{F}L^{\prime }\\ \frac{L_{0}}{2-A^{F}}\;\mathrm{otherwise}\; \end{array}\right..$$

According to [29], when the number of SIC iterations is H and the normalized load is G, the throughput of CRDSA3 is:

(39) $$Tp\left(H|G\right)=G\left[1-{\left(1-p_{\mathrm{pd}}^{A}\left(H|G\right)\right)}^{3}\right],$$

where $p_{\mathrm{pd}}^{A}(H|G)$ is the probability of a replica $p^{A}$ of a packet being successfully transmitted:

(40) $$p_{\mathrm{pd}}^{A}\left(H|G\right)\leq p_{\mathrm{al}}^{A}\left(G\right)+2\sum_{i=1}^{GM-1}p_{\mathrm{int}}\left(i|G\right){\left[p_{\mathrm{pd}}^{A}\left(H-1|G\right)\right]}^{i},$$

while $p_{\mathrm{int}}(i|G)=C_{GM-1}^{i}{\left(3/M\right)}^{i}{\left(1-3/M\right)}^{GM-1-i}$ is the probability of a packet being interfered by i packets in a given slot, and $p_{\mathrm{al}}^{A}(G)=p_{\mathrm{int}}(0|G)=(1-3/M{)}^{GM-1}$ is the probability that the replica $p^{A}$ is alone in a given slot.

The normalized load of this frame is G=L/M. Therefore, when SIC iterates H times, the expected throughput of the frame is:

(41) $$E\left(Tp\left(H|G\right)\right)=\frac{L_{0}}{2A^{F}M}\left[1-{\left(1-p_{\mathrm{pd}}^{A}\left(H|\frac{L_{0}}{A^{F}M}\right)\right)}^{3}\right]+\frac{L_{0}}{2\left(2-A^{F}\right)M}\left[1-{\left(1-p_{\mathrm{pd}}^{A}\left(H|\frac{L_{0}}{\left(2-A^{F}\right)M}\right)\right)}^{3}\right].$$

CRDSA3 reaches maximum throughput at the critical load value $L_{0}$ (the normalized load is $G_{0}=L_{0}/M$):

(42) $$Tp_{\max}=Tp\left(H|G_{0}\right)=\frac{L_{0}}{M}\left[1-{\left(1-p_{\mathrm{pd}}^{A}\left(H|\frac{L_{0}}{M}\right)\right)}^{3}\right].$$

It can be observed that the higher the load forecast precision (i.e., the closer to 1), the closer the load after control will be to the critical value, and the closer the throughput will be to the maximum throughput. The expected value of the gap between the throughput and the maximum throughput caused by the load forecast error is:

(43) $$E\left(\Delta Tp\right)=E\left(Tp_{\max}-Tp\left(H|G\right)\right)=Tp_{\max}-E\left(Tp\left(H|G\right)\right).$$

## 4. Performance Evaluation

In this section, we test the performance of LC-CRDSA3 through a series of simulation experiments. The main experimental parameter settings are listed in Table 1. In our experiments, we focus on an uplink random access channel of a satellite. Each frame of this channel contains 1000 slots, each slot duration is 0.5 ms, and one packet can be transmitted in a single slot. A total of 2000 ground nodes send data to the satellite through this channel, and the one-way propagation delay between a node and the satellite is 40 ms. Among the 2000 nodes, 1000 nodes periodically generate packets, with the packet generation period of each node being randomly determined. Another 1000 nodes randomly generate packets, and the number of packets generated by each node in each slot obeys a Poisson distribution. The average generation rate of periodic and random packets is the same. Our experiments emulate transmitting these packets. In order to avoid a large number of packets accumulating in the network when the average data generation rate is high, we set the number of packet transmission opportunities to 6. In addition to the packet being transmitted or retransmitted, if the packet is not transmitted due to load control, it is considered to have used a transmission opportunity. Following a failed transmission, a packet randomly waits for 0–9 frames before attempting transmission again. The number of SIC iterations is 10. In load forecasting, we retrain the forecasting model (neural network or support vector machine) every 2000 frames. The neural network is a three-layer feedforward neural network with a hidden layer. The relevant parameters of neural networks and support vector machines were determined through experimental testing. The total simulation time is 8000 frames. Since load control was not performed on the first 2000 frames, the data from the 2001st frame to the 8000th frame were recorded as the experimental results.

We first tested the overall performance of LC-CRDSA3 and analyzed the load estimation and load forecasting performance. Next, we compared the LC-CRDSA3 scheme with CRDSA3.

### 4.1. Performance Analysis of LC-CRDSA3

LC-CRDSA3 improves the network throughput in a dynamic load environment through load control, for which load estimation and load forecasting form the basis. We first tested the overall performance of LC-CRDSA3, then tested the accuracy of load estimation and load forecasting, along with their impact on performance.

#### 4.1.1. Overall Performance

We tested the throughput of LC-CRDSA3 under different average data generation rates (hereinafter referred to as data rates), as shown in Table 2. Here, the data rate reflects the network load. We used both neural network and support vector machine to forecast load (NN and SVM respectively in Table 2); the number of historical load values used by both models for forecasting is 70. We set the number of neurons in the hidden layer of the neural network P=10; the maximum error allowed by support vector regression ε=0.01, the penalty factor *D* = 10, and the Gaussian kernel parameter σ=5. Overall, LC-CRDSA3 achieves good throughput performance at the tested data rates. When g≤0.6, the overall load is comparatively low. A small number of failed packets, or packets that have no chance of being transmitted due to load control, can be successfully transmitted in subsequent frames; thus, the throughput is equal to the data rate. When g≥0.7, through load control, LC-CRDSA3 can obtain throughput that approaches the theoretical maximum (about 0.7).

#### 4.1.2. Load Estimation Performance

In order to evaluate the effectiveness of the MLE-based load estimation algorithm (hereinafter the MLE algorithm) proposed in this paper, we compare it with the two algorithms in Equations (3) and (4) (hereinafter the Cha and Kha algorithms, respectively). In order to make the Cha and Kha algorithms applicable when the number of replicas r=3, we modify the load estimation expressions of the two algorithms as follows:

(44) $${\hat{L}}^{\mathrm{Cha}}=\frac{s+2.39c}{3},$$

(45) $${\hat{L}}^{\mathrm{Kha}}=\frac{1}{3}\arg\underset{L}{\min}\left|E\left(I\right)-i\right|=\frac{1}{3}\langle \frac{\mathrm{lg}\left(\frac{M}{i}\right)}{\mathrm{lg}\left(\frac{M}{M-1}\right)}\rangle .$$

In more detail, we first estimate the number of packet replicas, then get the number of packets. We first test the load estimation precisions of the three algorithms, then test the load forecast precision and throughput of LC-CRDSA3 when these three algorithms are used.

**Figure 3:**
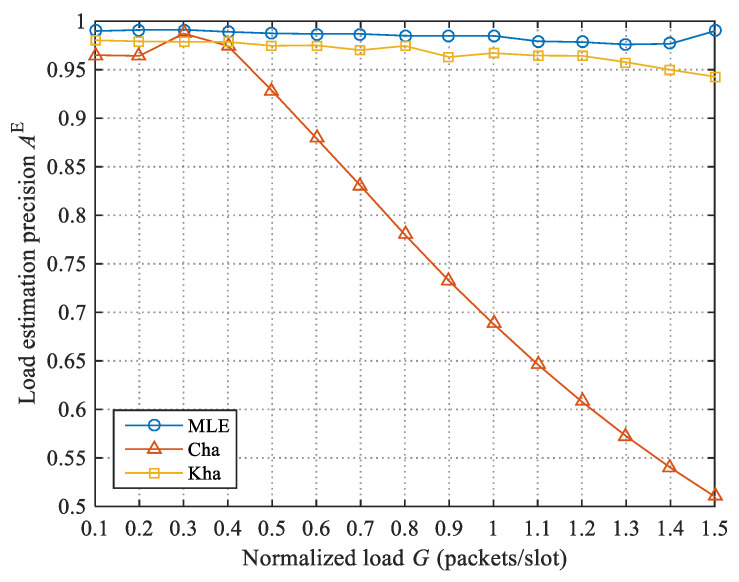
Comparison of load estimation precisions of the three load estimation algorithms.

Due to the load control, we cannot directly test the load estimation precisions of the three algorithms under different loads in LC-CRDSA3. We therefore test the load estimation precisions of the three algorithms under different normalized loads through additional simulation experiments. Every algorithm is tested 100 times under each normalized load; the average load estimation precision obtained is shown in Figure 3. Overall, the load estimation precision of MLE is higher than that of Cha and Kha, with an average increase of 33% and 1.77% respectively. Under the tested normalized load, both MLE and Kha obtain high load estimation precision. Although the ratio of increase relative to Kha is not large, MLE is more versatile and can be applied to different loads. Experimental tests show that when G≥1.9, Kha can no longer be applied, as there are almost no idle slots. In addition, when G>0.4, the load estimation precision of Cha drops sharply. Cha assumes that the number of packets in a collided slot is 2.39 on average; when the load is higher, the error of this assumption increases rapidly because there are more packets in a collided slot.

**Figure 4:**
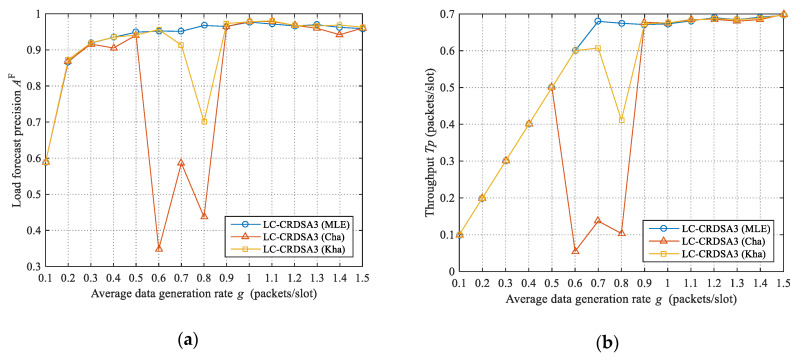
Load forecast precisions and throughput under the three load estimation algorithms: (**a**) Load forecast precisions under the three algorithms; (**b**) Throughput under the three algorithms.

We use the above three algorithms to estimate the load in LC-CRDSA3 in order to evaluate the impact of load estimation precision on load forecast precision and throughput, as illustrated in Figure 4. We use neural networks for load forecasting. When no idle slot exists, the Kha algorithm uses the Cha algorithm to estimate the load.

When g≤0.5, there is little difference between the load forecast precisions under the three algorithms; this is because the load is relatively low under this condition, with the result that the load estimation precisions of the three algorithms are relatively high. We can further observe that the lower the data rate, the lower the load forecast precisions of the three algorithms; this is because the load is more bursty when the data rate is low, making forecasting more difficult. Due to the low load, the throughputs of the three algorithms are equal to the data rate.

Interestingly, when g≥0.9, the load forecast precisions under the three algorithms are also both close and all relatively high. When the data rate is high, more packets are accumulated in the network, the original load becomes stable, and load forecasting is accordingly less difficult. Due to the load control, the actual load under the three algorithms is kept at a low level. Because the load estimation precisions of the three algorithms are relatively high when the load is low, and neural network-based load forecasting can tolerate a certain degree of noise, the load forecast precisions under all three algorithms are relatively high. Corresponding to the load forecast precision, the throughput is also relatively high for all algorithms.

When 0.5<g<0.9, the load forecast precisions under Cha and Kha are significantly lower than that of MLE. More specifically, the load forecast precision under MLE is improved by 119.08% and 13.91% on average relative to Cha and Kha respectively. Let’s analyze the reasons for the low load forecast precisions obtained by Cha and Kha. The load estimation precision affects the load forecast precision. Since the data rate is near the critical value, the frequency of load control is high. On the one hand, because the load under this circumstance is not too high, the load forecast precision of one frame will have a greater impact on the original load of subsequent frames, which will cause the original load to fluctuate significantly, increasing the difficulty of load forecasting. On the other hand, the load forecast precision will affect the actual load of the current frame. If the actual load is too high, this will in turn reduce the load estimation precision. Therefore, when the data rate is near the critical value, even a small difference in load estimation precision will have a substantial impact on load forecast precision. Corresponding to the load forecast precision, the throughput under MLE is 645.91% and 25.29% higher than that of Cha and Kha, respectively.

It can accordingly be seen that when the data rate approaches the critical load value, the load estimation precision has a significant impact on both load forecast precision and throughput: more specifically, the higher the load estimation precision, the higher the load forecast precision and throughput. In order to make full use of network resources, the data rate in a network is usually close to the critical load value. Improving the load estimation precision is thus crucial to improving throughput.

#### 4.1.3. Load Forecasting Performance

**Figure 5:**
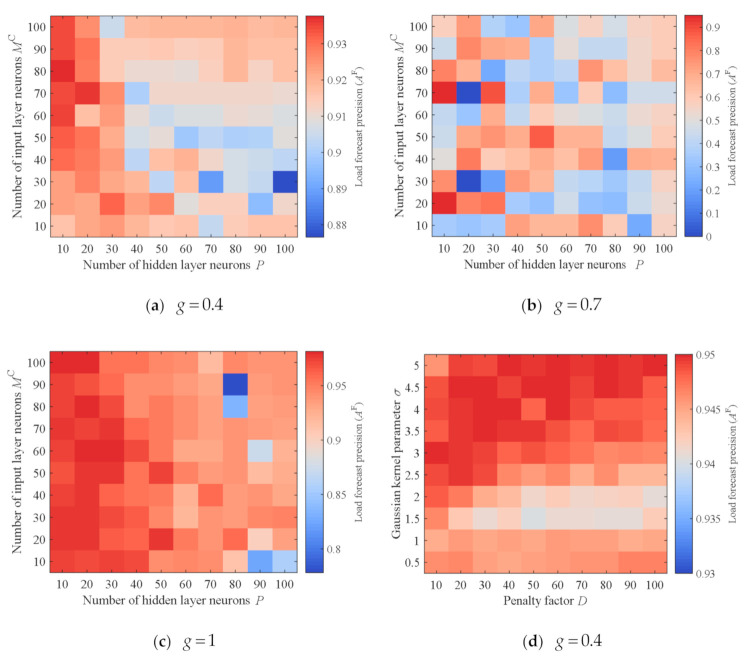

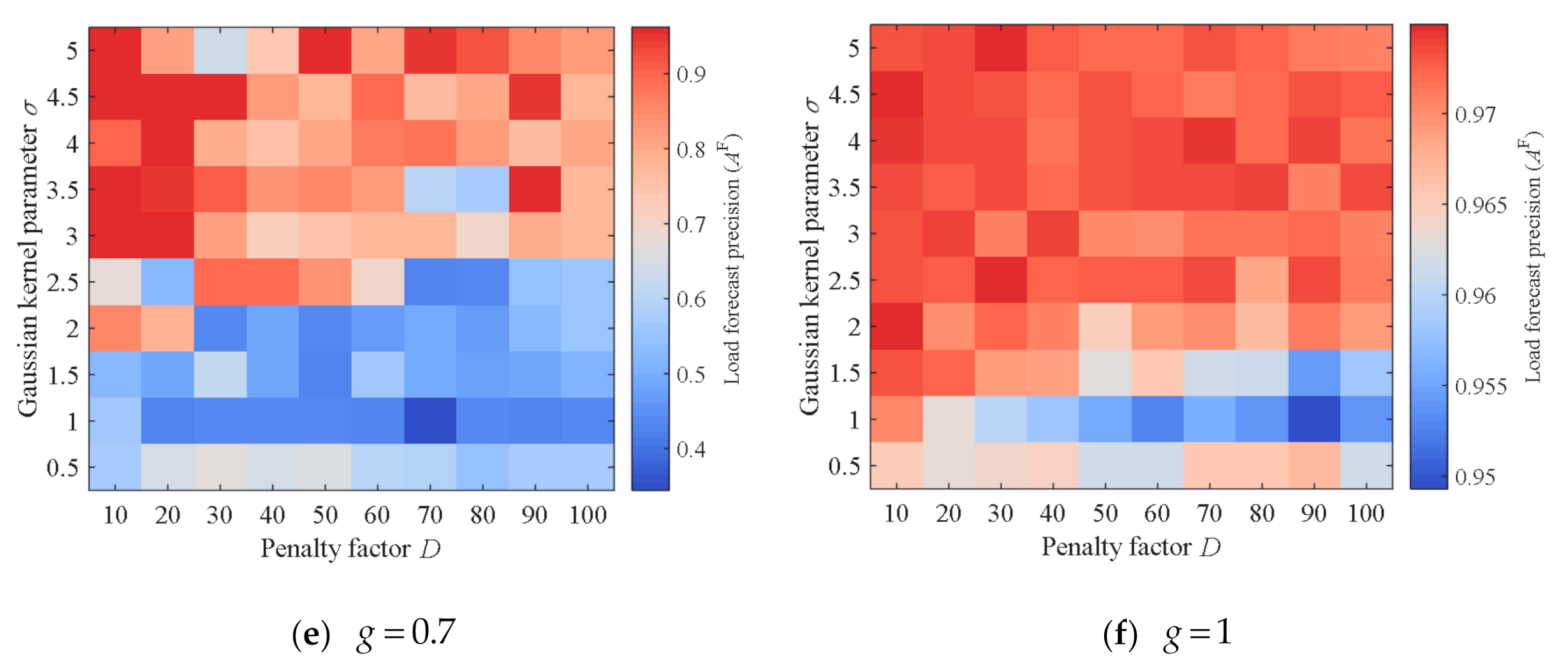
Load forecast precisions of neural network and support vector machine under different parameter combinations: (**a**) Load forecast precisions under different combinations of *P* and *M^C^* when g=0.4; (**b**) Load forecast precisions under different combinations of *P* and *M^C^* when g=0.7; (**c**) Load forecast precisions under different combinations of *P* and *M^C^* when g=1; (**d**) Load forecast precisions under different combinations of *D* and σ when g=0.4; (**e**) Load forecast precisions under different combinations of *D* and σ when g=0.7; (**f**) Load forecast precisions under different combinations of *D* and σ when g=1.

The load forecast precision directly determines the load control accuracy, which consequently affects throughput. It is necessary to select reasonable parameters when using a neural network or support vector machine for load forecasting. Below, we select the parameters of the neural network and the support vector machine through grid search. When using neural networks for load forecasting, two important parameters are the number of hidden layer neurons *P* and the number of historical load values used for prediction *M^C^* (that is, the number of input layer neurons). Moreover, the penalty factor *D* and the Gaussian kernel parameter σ are two important parameters for support vector machine-based load forecasting. In addition, the load forecast precision will also be affected by the data rate. In order to ensure that the selected parameters can maintain high load forecast precisions at different data rates, we select three representative data rates for testing: namely, 0.4 (below the critical load value), 0.7 (equal to the critical load value) and 1 (above the critical load value). We test the load forecast precisions under different combinations of *P* and *M^C^*, as well as different combinations of *D* and σ, at the three data rates; results are presented in Figure 5.

Overall, as can be seen from Figure 5a–c, when the number of hidden layer neurons is small, the neural network obtains higher load forecast precision. Across different time periods, the law of load changes will be different. When *P* is small, the neural network has a lower degree of fitting and also attains better generalization ability. When *P =* 10 and *M^C^* = 70, the neural network has high load forecast precisions under the three data rates (0.9348, 0.9515 and 0.9770 respectively). Hence, we choose them as the neural network parameters in the subsequent experiments.

With reference to the test results obtained by the neural network, we also set *M^C^* = 70 for the support vector machine. We further set the maximum error allowed for support vector regression to ε=0.01. As can be seen from Figure 5d–f, overall, when the penalty factor *D* is small, the support vector machine achieves higher load forecast precision; this is because the degree of fitting of the support vector machine is lower and it has better generalization ability under this condition. In addition, the larger the Gaussian kernel parameter σ, the higher the load forecast precision. When *D* = 10 and σ=5, the support vector machine attains high load forecast precisions under the three data rates (0.9457, 0.9620 and 0.9744, respectively). We accordingly choose them as the parameters of the support vector machine in the subsequent experiments.

In addition, we also observed that the load forecast precision at *g* = 0.7 is generally lower than at *g* = 0.4 and *g* = 1, regardless of whether neural network or support vector machine are used. This is because load forecasting is more difficult when *g* = 0.7, which also verifies our analysis in Section 4.1.2.

**Figure 6:**
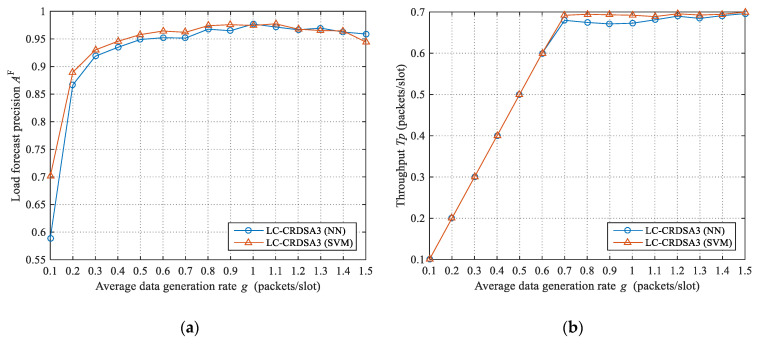
Load forecast precisions and throughput when using neural network and support vector machine for load forecasting: (**a**) Load forecast precisions under the two methods; (**b**) Throughput under the two methods.

The load forecast precisions and throughput when using neural network and support vector machine for load forecasting are plotted in Figure 6. Overall, under the tested data rates, higher load forecast precision and throughput is obtained by the support vector machine. More specifically, the load forecast precision of support vector machine is 1.86% higher on average than that of the neural network. Moreover, when *g* ≥ 0.7, the throughput of using support vector machine is 1.68% higher on average than that of using neural network.

### 4.2. Comparisons with Existing Scheme

In this section, we compare the LC-CRDSA3 scheme with the CRDSA3 scheme to test the impact on performance of introducing load control. In our experiment, all settings are identical across schemes except for the fact that CRDSA3 does not perform load control. LC-CRDSA3 uses neural network and support vector machine for load forecasting respectively. We compare the throughput, packet loss rate and packet delay.

#### 4.2.1. Throughput

**Figure 7:**
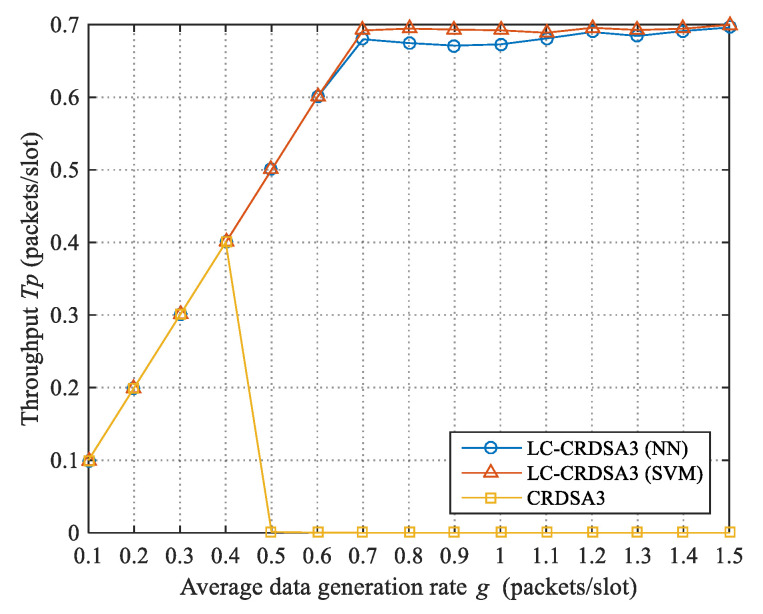
Comparison of throughput between LC-CRDSA3 and CRDSA3.

The throughput of LC-CRDSA3 and CRDSA3 at different data rates g is presented in Figure 7. When *g* ≤ 0.4, the throughput of these two schemes is equal to the data rate due to the low load. When *g* > 0.4, the failed and newly generated packets in CRDSA3 cause the packets in the network to accumulate continuously; therefore, the load of CRDSA3 is far above the critical value, and the throughput quickly drops to zero. According to the test results, CRDSA3 obtains the maximum throughput of 0.48 when the data rate is 0.48, which is far lower than the theoretical maximum (about 0.7). Due to load control, LC-CRDSA3 can still maintain a high throughput at a high data rate. When using support vector machine and g=1.5, LC-CRDSA3 obtains the maximum throughput of 0.6998, which is 45.79% higher than the maximum throughput of CRDSA3.

#### 4.2.2. Packet Loss Rate

**Figure 8:**
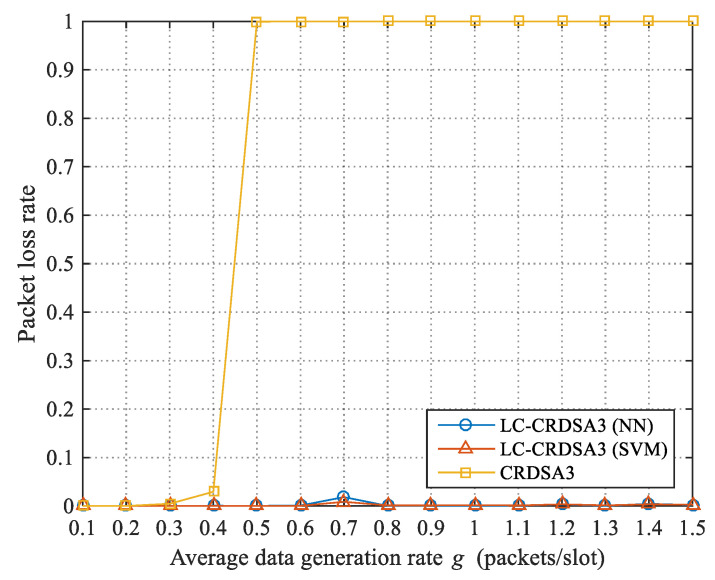
Comparison of packet loss rates between LC-CRDSA3 and CRDSA3.

In LC-CRDSA3, some packets have no opportunities for transmission due to load control. We define the packet loss rate of a frame as the ratio of the number of failed packets to the total number of transmitted packets. The packet loss rate of the two schemes under different data rates *g* is plotted in Figure 8. When *g* ≤ 0.4, the packet loss rate of the two schemes is relatively low, since the probability of data collision is also low. When *g* > 0.4, the data collision probability of CRDSA3 increases sharply due to the increase in load, and the packet loss rate rapidly approaches 1. However, LC-CRDSA3 accurately controls the load, and keeps the load at a low level, meaning that the packet loss rate is always relatively low. Under the tested data rates, the average packet loss rates of LC-CRDSA3 using neural network and support vector machine are $2.19\times 10^{-3}$ and $1.73\times 10^{-3}$ respectively.

#### 4.2.3. Packet Delay

**Figure 9:**
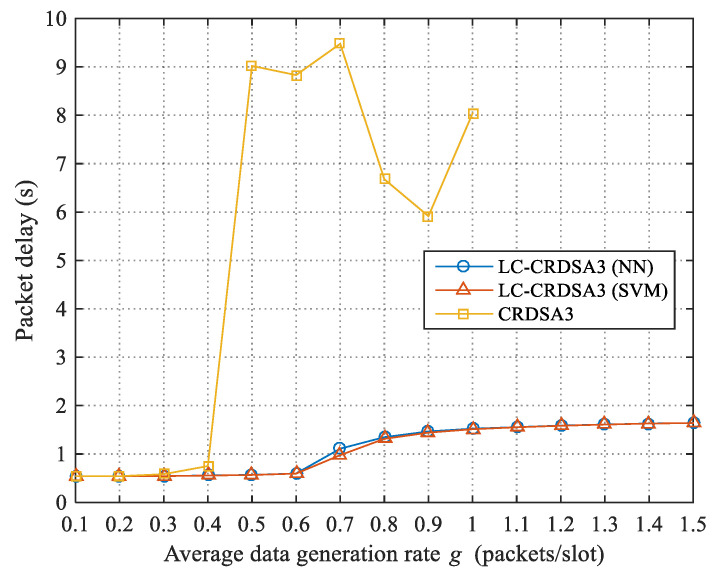
Comparison of packet delay between LC-CRDSA3 and CRDSA3.

We define packet delay as the time between a successfully transmitted packet being generated and being received by the satellite. The packet delay of LC-CRDSA3 and CRDSA3 under different data rates *g* is presented in Figure 9. When g≤0.4, the load is low, packets can be transmitted quickly, and the packet delay of CRDSA3 is relatively short; when *g* > 0.4, however, because the packet loss rate increases sharply, the number of packet retransmissions in CRDSA3 also increases, and the packet delay spikes rapidly. We can further observe that when 0.5≤g≤1, the packet delay of CRDSA3 undergoes substantial fluctuations; this is because the packet loss rate is too high and the number of successfully transmitted packets is very small, which causes the packet delay to exhibit a certain randomness. When g≥1.1, CRDSA3 can no longer achieve successful packet transmission because the load is too high.

Load control will increase the time a packet spends waiting for transmission. When the data rate is large, a packet will need to wait longer for a transmission opportunity. The packet delay of LC-CRDSA3 therefore increases gradually as the data rate increases. At the test data rate, the average packet delays of LC-CRDSA3 using neural network and support vector machine are 1121.44 ms and 1106.43 ms, respectively.

From the experimental results, it can be observed that LC-CRDSA3 significantly improves on the performance of CRDSA3 in terms of throughput, packet loss rate and packet delay by introducing load control.

## 5. Conclusions and Future Work

Random access is one of the most competitive multiple access schemes in the future S-IoT context due to its support for massive connections and grant-free transmission, as well as its ease of implementation, all of which constitute significant advantages. However, existing random access schemes are highly sensitive to load; once the load exceeds a certain critical value, the throughput will sharply decline. Moreover, the network load of S-IoT changes dynamically owing to the variable satellite coverage and bursty traffic. This makes the actual throughput of existing random access schemes is far below the theoretical maximum when these schemes are directly applied to S-IoT. Accordingly, in this paper, we propose LC-CRDSA3, an intelligent load control-based random access scheme. LC-CRDSA3 actively controls the network load. When the load threatens to exceed the critical value, only certain nodes are allowed to send data. In order to accurately implement load control, LC-CRDSA3 first estimates the load of each frame through the proposed MLE-based load estimation algorithm, then uses computational intelligence-based time series forecasting technology to predict the load of future frames. We tested the LC-CRDSA3 via a series of simulation experiments, which verify the accuracy of its load estimation and load forecasting. Moreover, compared with CRDSA++, LC-CRDSA3 can obtain network throughput that is close to the theoretical maximum across a wide load range in an S-IoT context where the load is dynamically changing.

In future work, we will conduct additional research into multiple access technology in the S-IoT environment. In terms of load forecasting, we will explore other time series forecasting methods, such as Bayesian networks, to further improve the load forecast precision. In particular, the network load of S-IoT will change drastically in both small time scales (such as slots and frames) and large time scales (such as minutes and hours); thus, we plan to adopt wavelet analysis and other methods to forecast load from the perspective of multiple time scales. We will also consider service priority and quality of service requirements during load control, which will enable high-priority and delay-sensitive services to first obtain transmission opportunities. In addition, we could consider optimizing multiple access for different service types (for example, semi-persistent scheduling can be used for periodic services).

## Figures and Tables

**Table 1:** Experimental parameter settings.

Parameter	Value
Number of slots in each frame	1000
Duration of each slot	0.5 ms
Number of nodes	2000
One-way propagation delay between node and satellite	40 ms
Number of packet transmission opportunities	6
Number of frames waiting to participate in retransmission	0~9
Number of frames between forecasting model training	2000
Simulation time	8000 frames

**Table 2:** Throughput of LC-CRDSA3 at different average data generation rates.

g	**0.1**	**0.2**	**0.3**	**0.4**	**0.5**	**0.6**	**0.7**	**0.8**
LC-CRDSA3 (NN)	0.1000	0.2000	0.3000	0.4000	0.5000	0.6000	0.6798	0.6746
LC-CRDSA3 (SVM)	0.1000	0.2000	0.3000	0.4000	0.5000	0.6000	0.6920	0.6945
g	**0.9**	**1**	**1.1**	**1.2**	**1.3**	**1.4**	**1.5**	
LC-CRDSA3 (NN)	0.6711	0.6728	0.6809	0.6898	0.6844	0.6912	0.6960	
LC-CRDSA3 (SVM)	0.6931	0.6922	0.6888	0.6956	0.6926	0.6944	0.6998	

## Data Availability

The data presented in this study are available on request from the authors.
